# Porphyrin accumulation induced by 5-aminolaevulinic acid esters in tumour cells growing *in vitro* and *in vivo*

**DOI:** 10.1038/sj.bjc.6600460

**Published:** 2002-07-02

**Authors:** R G Tunstall, A A Barnett, J Schofield, J Griffiths, D I Vernon, S B Brown, D J H Roberts

**Affiliations:** Centre for Photobiology and Photodynamic Therapy, University of Leeds, Leeds LS2 9JT, UK

**Keywords:** aminolaevulinic, esters, protoporphyrin-IX, photodynamic, PDT

## Abstract

The ability of 5-aminolaevulinic acid and some of its esterified derivatives to induce porphyrin accumulation has been examined in CaNT murine mammary carcinoma cells growing in culture and as tumours *in vivo*. Topical or intravenous administration of 5-aminolaevulinic acid-esters to mice bearing subcutaneous tumours produced lower porphyrin levels in the tumour than an equimolar dose of 5-aminolaevulinic acid. Reducing the dose of intravenous hexyl- or benzyl-ALA and topical hexyl-5-aminolaevulinic acid resulted in a dose-dependent reduction in porphyrin accumulation. A number of normal tissues accumulated higher concentrations of porphyrins than tumour tissue following intravenous administration of 5-aminolaevulinic acid-esters. Esterase activity in these normal tissues was greater than that in tumour tissue. In contrast to the situation *in vivo*, all of the 5-aminolaevulinic acid-esters examined were at least as effective as 5-aminolaevulinic acid when applied to cloned CaNT cells *in vitro*, with the drug concentration required for maximum porphyrin accumulation varying with ester chain-length. Tumour cells growing in culture released esterase activity into the medium. These findings suggest that the efficacy of 5-aminolaevulinic esters may vary depending on the esterase activity of the target tissue, and suggest caution when interpreting the findings of *in vitro* studies using these and similar prodrugs.

*British Journal of Cancer* (2002) **87**, 246–250. doi:10.1038/sj.bjc.6600460 www.bjcancer.com

© 2002 Cancer Research UK

## 

Photodynamic therapy (PDT) is increasingly being used for the treatment of many malignant and pre-malignant conditions. Although the photosensitising drug Photofrin-II® has received regulatory approval for use in a number of conditions (Dougherty *et al*, 1998), patients often have to be limited to subdued lighting for several weeks following treatment due to its prolonged retention in the skin (Carruth, 1998). In contrast, skin photosensitivity following the systemic administration of 5-aminolaevulinic acid (ALA), a metabolic precursor of the photosensitiser protoporphyrin-IX (PpIX), is relatively mild and short-lived (Rick *et al*, 1997). ALA-based PDT is now being investigated as a treatment for several conditions (Roberts and Cairnduff, 1995; Peng *et al*, 1997b).

The hydrophilic nature of ALA (Novo Rodriguez *et al*, 1995) may limit its ability to penetrate through skin or cell membranes (Peng *et al*, 1997a) and thereby restrict its topical use to the treatment of superficial disease. As a result, esterified derivatives of ALA, which are less hydrophilic than the parent compound (Uehlinger *et al*, 2000), are under investigation as possible alternatives to ALA. Work *in vitro* has demonstrated that a number of cell lines have the ability to take-up and metabolise certain ALA-esters into PpIX at a faster rate, and at lower concentrations, than ALA (Kloek and Beijersbergen van Henegouwen, 1996; Peng *et al*, 1996). A number of *in vivo* studies have suggested that topical application of some ALA esters can result in greater PpIX accumulation than similar concentrations of ALA (Peng *et al*, 1996; Lange *et al*, 1999; van den Akker *et al*, 2000). In contrast, however, clinical work with human solar keratoses showed methyl-ALA to induce less PpIX than ALA (Fritsch *et al*, 1998).

ALA has been shown to be effective at inducing PpIX after oral, intravenous (i.v.) or topical application *in vivo* (Cairnduff *et al*, 1995; Peng *et al*, 1995; van den Boogert *et al*, 1998). As yet, no data regarding the systemic administration of ALA-esters have been published. The aim of this study was to investigate the induction of porphyrins by ALA and ALA-esters in mouse mammary carcinoma cells growing both *in vitro* and as tumours *in vivo*.

## MATERIALS AND METHODS

### Chemicals

ALA hydrochloride was synthesised using a method based on that of Pfaltz and Anwar (1984), purified with active charcoal and re-crystallised from acetone. Hydrochloride salts of ethyl-, propyl-, butyl-, pentyl-, cyclohexyl-, hexyl- and benzyl-ALA were prepared using the method described by Kloek *et al* (1998). Elemental analysis showed them to be at least 90% pure. Unguentum Merck® emollient cream was obtained from Merck Ltd, Lutterworth, UK. All other chemicals were obtained from BDH Laboratory Supplies, Poole, UK and Sigma-Aldrich Co. Ltd., Poole, UK.

### Tumour and animals

The CaNT tumour is a poorly-differentiated non-immunogenic carcinoma, probably of mammary origin, which arose spontaneously in a female CBA/Gy mouse (Hewitt *et al*, 1973). Tumour and initial breeding pairs of CBA/Gy mice were kindly provided by Dr DJ Chaplin and Mr P Russell respectively (CRC Gray Laboratories, Middlesex, UK). Mice were housed in a 12 h light/dark cycle and given free access to water and food (B&K Ltd, Hull, UK, Universal Standard Rodent Diet). Once ALA or ALA-ester had been administered, animals were maintained in subdued lighting. All work was carried out in accordance with the UKCCCR guidelines for use of animals in experimental neoplasia (UKCCCR 1998).

Tumours were maintained by serial transplantation for a maximum of 10 passages. For experiments, tumour fragments of approximately 5 mm^3^ were implanted subcutaneously (s.c.) into the flanks of 6–10-week-old mice (21–27 g), and allowed to grow to 150–250 mm^3^ prior to randomisation to control or treatment groups.

### Cells and cell culture

CaNT cells were harvested from tumour explants growing at 37°C in Dulbecco's minimal essential medium (DMEM) containing 10% foetal calf serum (Life Technologies, Paisley, UK) in an atmosphere of 5% CO_2_ in air. Clones were prepared by dilution and subsequently shown to be tumorigenic by s.c. inoculation into CBA/Gy mice. Cells were routinely passaged using 1% trypsin/EDTA (Life Technologies, Paisley, UK).

### ALA and ALA-ester-induced generation of porphyrins in murine tissues

ALA (0.06–1.2 mmol kg^−1^) and ALA esters (0.0012–1.2 mmol kg^−1^) in 10 mM phosphate buffered saline (PBS, pH 7.4) were administered via a lateral tail vein. Animals were killed at intervals up to 24 h later, and tissue samples (0.1–0.2 g wet weight) snap-frozen in liquid nitrogen and stored at −85°C.

For topical application, ALA (20% w/w) and hexyl-ALA (0.02–20% w/w) were prepared in Unguentum Merck® emollient cream immediately prior to use. Skin overlying the tumour was shaved and tape-stripped and 0.2 ml cream rubbed into the skin for two minutes with a gloved finger. Control animals received 0.2 ml cream alone. Tumour tissue was collected 1–6 h later as described above.

Porphyrin concentrations were determined by a method modified from that of Longas and Poh-Fitzpatrick (1980). The porphyrin extraction efficiency of this assay has previously been calculated as 90% (Poh-Fitzpatrick and DeLeo, 1977). Briefly, tissue samples were incubated in 1 M aqueous NaOH in the dark at 40°C for 16 h and porphyrins extracted into 4 : 1 ethyl acetate : acetic acid. The organic layer was removed and porphyrins extracted into 1 M aqueous HCl. Porphyrin fluorescence in the resultant aqueous layer was measured with a Kontron SFM25 fluorimeter (Kontron Instruments Ltd, Watford, UK; excitation 403 nm, emission 602 nm). A solution of haematoporphyrin was used as fluorescence standard. Data from tissues treated with ALA- or ALA-ester were corrected by subtracting the porphyrin concentration in equivalent untreated tissues.

### ALA and ALA-ester-induced generation of porphyrins in cultured cells

Cloned CaNT cells were grown in 24-well plates until 50–75% confluent. After thorough washing in warm PBS, cells were incubated in the dark for 3 h with ALA or ALA-esters in serum-free DMEM at pH7.4. Cells were then washed twice in PBS and solubilised in the dark in 0.5 ml of 26.5 M formic acid for 15 min at room temperature. Porphyrin fluorescence was determined as described above. Cellular protein concentration was determined in duplicate wells using the BioRad protein microassay (Biorad Laboratories Ltd, Hemel Hempstead, UK). Porphyrin analyses were performed in quadruplicate, and all experiments were repeated at least twice. Data from cells treated with ALA- or ALA-ester were corrected by subtracting the porphyrin concentration in equivalent untreated cells.

### HPLC analysis of porphyrins

Porphyrins in tissue samples or cultured cells were extracted as described for fluorimetry and the pH adjusted to 5 or above with saturated sodium acetate. The samples were then further extracted into ethyl acetate, washed with an equal volume of distilled water and passed through a filter paper saturated in ethyl acetate. They were then dried under a stream of nitrogen gas and stored in the dark at 4°C until required.

Dry porphyrin samples were dissolved in 100 μl of methanol containing 10% HCl immediately before HPLC analysis. Reverse phase HPLC analysis of 20 μl of reconstituted sample was performed using a Waters Nova Pak C18 analytical column (150 mM×3.9 mm). Solvent A was 10% acetonitrile/90% ammonium acetate (1 M, pH 5.16). Solvent B was 90% methanol/10% acetonitrile. Fluorescence detection was performed at 400 nm excitation and 620 nm emission. Sample absorbance was measured at 400 nm. Standards solutions of known porphyrins were used to identify the porphyrins in samples.

### Non-specific esterase assay

Non-specific esterase activities in tissue homogenates, lysates of cultured cells and in cell-conditioned or fresh culture medium were determined using an adaptation of the fluorescein diacetate method described by Washbrook and Riley (1997).

### Statistical analysis

Statistical analyses were performed using SPSS v9.0 for Windows. The independent-samples Student *t*-test was used to test the difference between means. Normality of data was confirmed using the Kolmogorov-Smirnov statistic or Q-Q normal probability plot.

## RESULTS

### ALA and ALA-ester-induced generation of porphyrins in murine tissues

**Figure 1:**
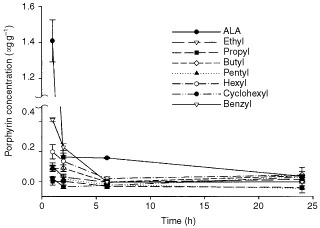
Porphyrin concentration in s.c. CaNT tumours following i.v. administration of 0.12 mmol kg^−1^ ALA or its esterified derivatives. Data are means±s.e. (*n*=4).

Porphyrin levels in the CaNT tumour were highest 1 h after i.v. administration of ALA or ALA-esters (0.12 mmol kg^−1^), except in the cases of propyl- and cyclohexyl-ALA which did not induce porphyrin accumulation in tumour tissue (Figure 1

). ALA induced higher porphyrin levels than any of its esters (*P*<0.001).

Reducing the dose of i.v. administered ALA, hexyl-ALA or benzyl-ALA resulted in a dose-dependent reduction in the concentration of porphyrins accumulating in the tumour (Table 1

**Table 1:**
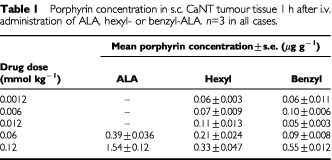
Porphyrin concentration in s.c. CaNT tumour tissue 1 h after i.v. administration of ALA, hexyl- or benzyl-ALA. *n*=3 in all cases

).

**Figure 2:**
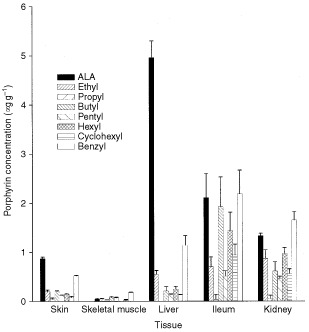
Porphyrin concentration in selected normal mouse tissues 1h after i.v. administration of 0.12 mmol kg^−1^ ALA or its esterified derivatives. Data are means±s.e. (*n*=4).

In normal murine tissues, the ability of i.v. ALA and its esters (0.12 mmol kg^−1^) to induce porphyrin accumulation was tissue-dependent (Figure 2

). In skeletal muscle, porphyrin levels were generally low, although benzyl-ALA was most effective. In skin and liver, ALA generated the highest porphyrin levels, whereas in the ileum, ALA, butyl-ALA and benzyl-ALA were similarly effective. In the kidney, benzyl-ALA and ALA generated similar porphyrin levels (0.12>*P*>0.05). As in the tumour, propyl-ALA failed to induce porphyrin accumulation in any of the normal tissues examined.

**Figure 3:**
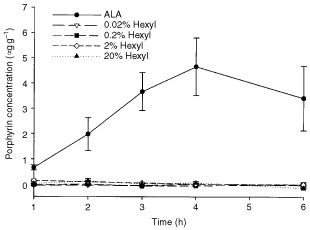
Porphyrin concentration in s.c. CaNT tumours following topical application of emollient cream containing ALA (20% w/w) or hexyl-ALA (0.02–20% w/w). Data are means±s.e. (*n*=4).

Topical application of 20% ALA cream induced porphyrin accumulation in the CaNT tumour, peaking 4 h after administration (Figure 3

). In contrast, 0.02–20% hexyl-ALA induced little or no porphyrin accumulation in tumour tissue.

### ALA and ALA-ester-induced generation of porphyrins in cultured cells

**Figure 4:**
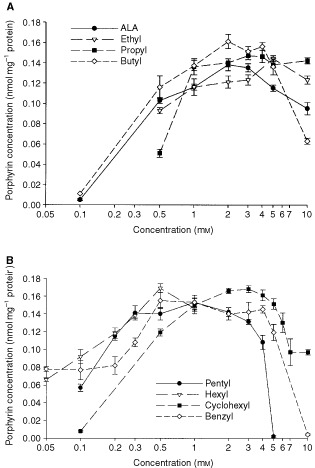
Porphyrin concentration in cultured CaNT cells after 3 h incubation in serum-free medium containing ALA or (**A**) elthyl-, propyl- or butyl-ester (**B**) pentyl-, hexyl-, cyclohexyl- or benzyl-ester. Data are means±s.e. (*n*=8).

All ALA-esters induced intracellular accumulation of porphyrins in tumour cell clones (Figure 4a,b

). When esters were administered at their optimum concentrations, the resulting porphyrin levels were similar to or above that induced by the optimum concentration of ALA. When pentyl-, hexyl- and benzyl-ALA were administered above concentrations of 6 mM, 1 mM and 10 mM respectively, cells became rounded and detached from the substratum. This was not seen for any other ALA-esters at the concentrations investigated.

### HPLC analysis of porphyrins induced by ALA and ALA-esters

Following i.v. administration of ALA at 0.12 mmol kg^−1^, PpIX accounted for an average of 88% of the porphyrins in tumour tissue, 98% in skin, 97% in skeletal muscle, 80% in liver, 87% in ileum, and 89% in kidney. Following *in vivo* administration of ALA-esters at 0.12 mmol kg^−1^, PpIX accounted for 83–100% of the porphyrins generated by murine ileum depending on the ester applied. When ALA or ALA-esters were used at their optimum concentrations *in vitro,* PpIX accounted for 94–100% of porphyrins generated.

### Esterase activity in murine tissues and cultured cells

Non-specific esterase activity varied between the different tissues examined (Table 2

**Table 2:**
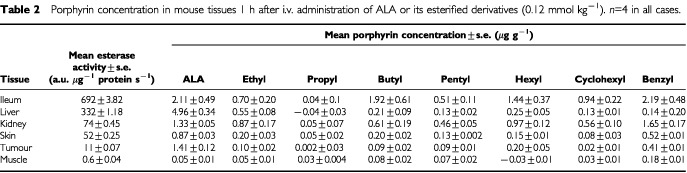
Porphyrin concentration in mouse tissues 1 h after i.v. administration of ALA or its esterified derivatives (0.12 mmol kg^−1^). *n*=4 in all cases

). In most cases, tissues with greater esterase activity accumulated a higher concentration of porphyrin following i.v. ester administration.

The mean esterase activity in cultured CaNT cells was 3.0±0.02 arbitary units/μg protein/s. In addition, serum-free DMEM exposed to cells for 3 h exhibited a mean esterase activity of 946±7.7 a.u./ml/s (value corrected for background activity in the medium of 59±16 a.u./ml/s).

## DISCUSSION

I.v. administration of ALA caused the accumulation of porphyrins in CaNT tumour growing s.c. and in a variety of normal murine tissues (Figures 1 and 2). Similarly, topical application of ALA to the skin induced porphyrin accumulation in underlying tumour (Figure 3). These results are in agreement with those of similar previous studies (Cairnduff *et al*, 1995; de Bruijn *et al*, 1999). In all cases, the predominant porphyrin species formed was protoporphyrin-IX.

In contrast, i.v. or topical administration of a range of ALA-esters had relatively little or no effect on the porphyrin concentration in the s.c. CaNT tumour. However, when given i.v., all the ALA-esters examined, with the exception of propyl-ALA, were able to induce porphyrin accumulation in a variety of normal tissues (Figure 2). This suggests that at least some murine tissues have the capacity to utilise certain ALA-esters for porphyrin synthesis, and that the results obtained in tumour tissue are not a reflection of murine metabolism *per se*.

Previous work *in vitro* has suggested that a number of ALA-esters, including hexyl-ALA, are most effective when used at much lower doses than ALA (Kloek *et al*, 1998; Lange *et al*, 1999; Uehlinger *et al*, 2000). Here, we found that reducing the dose of i.v. hexyl- or benzyl-ALA resulted in a dose-dependent reduction in tumour porphyrin levels to that of untreated controls. Reducing the dose of topical hexyl-ALA had no consistent effect on the very low levels of porphyrins observed in tumours up to 6 h after application. These findings strongly suggest that the relative inability of ALA-esters to induce porphyrin accumulation in tumour tissue compared to ALA was not the result of the esters being applied at inappropriately high concentrations.

In contrast to the results with CaNT tumours, all ALA-esters examined were able to induce similar or greater porphyrin concentrations than ALA in CaNT cells growing *in vitro* (Figure 4a,b). The optimum concentration of each ALA-ester was related to the length of the ester-linked chain, with longer-chain compounds inducing maximum porphyrin levels at lower concentrations than the shorter-chain derivatives. These findings are in broad agreement with previous observations (Gaullier *et al*, 1997; Kloek *et al*, 1998; Uehlinger *et al*, 2000).

It is not clear why the ability of ALA-esters to induce porphyrin accumulation differed between CaNT cells in culture and those growing as tumours *in vivo*. The observation that some non-fatty normal murine tissues were able to utilise at least some of the ALA-esters, including those with the longest carbon chains, suggests reduced bioavailability via sequestration into lipid-rich tissues is unlikely to explain the lack of effect in tumour tissue.

An alternative explanation is that introducing the tumour cells to tissue culture modified their metabolism such that ALA-esters could be more readily utilised. Up-regulation of intracellular non-specific esterase activity could, for example, facilitate ester hydrolysis and hence utilisation in porphyrin biosynthesis. With the exception of liver, there was a tendency for tissues with higher esterase activity to accumulate higher porphyrin concentrations following i.v. ALA-ester administration (Table 2). Although esterase activity in the cultured CaNT cells was relatively low, such cells released esterase activity into the surrounding medium. It is possible that pre-processing of ALA-esters whilst still in the extra-cellular milieu could have made ALA available to the cells *in vitro* in a way that may not have been available *in vivo*. Uptake of ALA-ester destined for intra-cellular cleavage at the same time as ALA derived from extra-cellular cleavage, may have resulted in greater ALA availability for porphyrin biosynthesis than could have been achieved by ALA-ester uptake alone. There is some evidence that ALA and at least some ALA-esters are taken into cells via different routes (Rud *et al*, 2000; Gederaas *et al*, 2001).

Clinical and animal studies do not yet provide a consistent answer with regard to the efficacy of ALA-esters (Peng *et al*, 1996; Lange *et al*, 1999; Fritsch *et al*, 1998; van den Akker *et al*, 2000). Here, we have found that several esters are less effective than ALA at inducing porphyrin accumulation in a s.c. mouse tumour. It is possible that the ability of ALA-esters to induce PpIX accumulation may be dependent on factors such as tumour type, metabolic status, or location, as well as on the actual ester applied. This may have significant implications for the clinical usefulness of this group of compounds. Whether the level of esterase activity present in the CaNT tumour is representative of that in other tumours, and whether other tumours would display the same differential response to ALA-esters applied *in vitro* and *in vivo*, remains to be determined however.

In conclusion, we have found that a number of ALA esters are at least as effective as ALA at inducing protoporphyrin IX accumulation in murine mammary carcinoma cells *in vitro*, but that the same compounds are either ineffective, or much less effective than ALA when administered *in vivo*. The greater complexity of the *in vivo* environment and/or culture-induced changes in cell properties may have been responsible for these differences. Such factors can limit the ability of *in vitro* test systems to predict drug effectiveness *in vivo*.
